# Platelet-Rich Plasma Supports Proliferation and Redifferentiation of Chondrocytes during *In Vitro* Expansion

**DOI:** 10.3389/fbioe.2017.00075

**Published:** 2017-12-06

**Authors:** Vivek Jeyakumar, Eugenia Niculescu-Morzsa, Christoph Bauer, Zsombor Lacza, Stefan Nehrer

**Affiliations:** ^1^Centre for Regenerative Medicine and Orthopedics, Danube University Krems, Krems an der Donau, Austria; ^2^OrthoSera GmbH, Krems an der Donau, Austria

**Keywords:** chondrocytes, dedifferentiation, autologous chondrocyte implantation, platelet-rich plasma, hyperacute serum

## Abstract

Articular cartilage regeneration is insufficient to restore sports injuries or defects that can occur from trauma. Treatment options for cartilage repair include autologous chondrocyte implantation (ACI) by isolation, expansion, and reimplantation of healthy donor chondrocytes. Chondrocyte expansion onto 2D substrates leads to dedifferentiation and loss of the cellular phenotype. We aimed to overcome the state of dedifferentiation by biochemical stimuli with platelet derivatives such as platelet-rich plasma (PRP) and hyperacute serum (HAS) to achieve sufficient cell numbers in combination with variable oxygen tension. Human articular chondrocytes from osteoarthritic (OA) cartilage chondrocytes were switched from 10% FCS supplementation to either 10% PRP or 10% HAS after initial passaging for further experiments under normoxic (20% O_2_) or hypoxic (1% O_2_) conditions. An XTT assay measured the effect of PRP or HAS on the cell proliferation at 3, 6, and 9 days. The chondrogenic redifferentiation potential of dedifferentiated chondrocytes was determined with reverse transcriptase quantitative real-time PCR for markers of expression for type II collagen (COL2A1), type I collagen (COL1A1), and matrix metalloproteinases MMP3, matrix metalloproteinase 13 (MMP13) at 24 and 72 h. Measured protein levels of 100% PRP or HAS by multiplex quantification revealed basic fibroblast growth factor, G-CSF, and PDGF were significantly higher in PRP than in HAS (*p* < 0.05) but LEPTIN levels did not differ. The quantified protein levels did not differ when isolated from same donors at a different time. Chondrocyte proliferation indicated that supplementation of 10% HAS enhanced the proliferation rate compared to 10% PRP or 10% FCS at 6 and 9 days significantly (*p* < 0.05). mRNA levels for expression of COL1A1 were significantly downregulated (*p* < 0.05) when cultured with 10% PRP than 10% HAS or 10% FCS under normoxic/hypoxic conditions. COL2A1 was significantly upregulated (*p* < 0.05) in PRP than 10% HAS or 10% FCS. MMP3 expression was downregulated after 72 h under all conditions. MMP13 was upregulated with 10% PRP at both 24 and 72 h but significantly downregulated under hypoxia (1% O_2_) for all circumstances. While HAS has its effect on chondrocyte proliferation, PRP enhances both proliferation and redifferentiation of dedifferentiated chondrocytes. PRP can replace standard usage of FCS for chondrogenic priming and expansion as implications for clinical use such as ACI procedures.

## Introduction

Articular cartilage is an avascular, alymphatic, aneural tissue that functions as a lubricating and load-bearing surface of the joint limited by the regenerative potential of its individual cell type, the chondrocyte. A defect at the site of tissue can often lead to larger lesions and without prompt treatment can result in a degenerative disorder such as osteoarthritis (OA). Autologous chondrocyte implantation (ACI) predominantly treats focal chondral defects ≥3–4 cm^2^ covered by periosteum layer (ACI-P) (Minas, 2001), collagen type I/III membrane (ACI-C) (Niemeyer et al., 2008), or the use of fibrin or hyaluronan matrix-assisted chondrocyte implantation (ACI-M/MACI) (Nehrer et al., 2008). ACI is a technique that involves isolation of autologous articular chondrocytes from a non-load-bearing site of the joint by enzymatic digestion, expanded, and implanted into the defect site (Peterson et al., 2000). Isolation of chondrocytes from the tissue biopsy involves enzymatic digestion which further dissociates the chondrocytes from their pericellular microenvironment (chondrons) that dedifferentiates the cells by losing their native chondrogenic phenotype (Vonk et al., 2014). Despite tremendous clinical success with ACI, there still exist areas for continued development of efficient protocols toward priming hyaline cartilage formation.

During *in vitro* expansion for ACI, articular chondrocytes are inclined to dedifferentiate toward a fibroblast-like phenotype by reducing expression of collagen type II (COL2A1) and increasing expression of collagen type I (COL1A1) at both the mRNA and protein levels (Schnabel et al., 2002). The dedifferentiation process has been indicated to progress over several passages in monolayer culture limiting the opportunity to obtain surmountable quantities of cells for transplantation in a differentiated state (Kang et al., 2007). The process of dedifferentiation can be overcome by supplementing culture medium with exogenous growth factors (Stewart et al., 2000; Pei et al., 2002) or culturing in biomaterials such as alginate or collagen type I and/or type II (hyaluronan) (Nehrer et al., 1997; Stevens et al., 2004; Mesenchymal, 2009). Redifferentiation of chondrocytes *in vitro* requires a multifaceted approach utilizing biochemical stimuli involving recombinant growth factors such as transforming growth factor-β, insulin-like growth factor-1, fibroblast growth factor-2, and platelet-derived growth factor BB (PDGF-BB) (Martin et al., 1999; Brandl et al., 2010). Biophysical stimuli include oxygen tension or mechanical loading (Kawanishi et al., 2007; Duval et al., 2009). Chondrocytes cultured on monolayers under low-oxygen tension maintains an oxidative phenotype (Heywood and Lee, 2010) and post-expansion when aggregated in 3D cultures begin to express elevated levels of collagen type II expression (Egli et al., 2008; Henderson et al., 2010). Hypoxic culture condition has been shown to enhance markers of redifferentiation, increased matrix formation concurrently inhibiting hypertrophic markers in both articular and osteoarthritic-derived chondrocytes (Markway et al., 2013). Disadvantages over growth factor supplementation include their high-cost utility and the technical challenges associated with biomaterials. Alternative substitutes such as platelet derivative products constitute a source of growth enriching factors which is also advantageous for its cost effective, large scale manufacturing with high standardization for cell culture.

Platelet-rich plasma (PRP) has gained wide attention in the field of regenerative medicine and cell-based therapies as a human alternative replacing fetal bovine serum for cell propagation. The α-granules in platelets constitute a natural source of growth factors and cytokines. Platelets can be ruptured either by repeated freeze–thaw cycles or activated by the addition of bovine thrombin to release the growth factor, cytokines from their granules referred in as platelet lysates (PL). The effects of both PRP and PL have been reported virtually by several *in vitro* studies on proliferation and differentiation of chondrocytes (Akeda et al., 2006; Drengk et al., 2009; Spreafico et al., 2009). In patients with knee joint degeneration, intra-articular injections of PRP injection of PRP seems to be a viable treatment for knee OA and has the potential to lead to symptomatic relief for up to 12 months (Smith, 2016). There appears to be an increased risk of local adverse reactions after multiple PRP injections but diminishes eventually (Filardo et al., 2015). Similarly, platelet-rich fibrin (PRF), a second-generation platelet derivative that can be formed by a natural coagulation process without any anticoagulants or biochemical modification (Dohan et al., 2006) is of interest for its hyperacute serum (HAS). HAS is separated from the blood cells during the PRF clotting phase within 10 min, and this is why HAS contains only the cytokines which are released in a hyperacute phase of clot activation. Moreover, HAS is beneficial over PRP due to its relatively simple activation of platelets without the need for exogenous thrombin addition.

The technical requirement of ACI pose a paradoxical challenge that the harvested mature chondrocytes need to get dedifferentiated to proliferate, then, when a sufficient number of cells obtained, they need to redifferentiate *in situ* to form a new cartilage layer. Therefore, culture conditions of articular chondrocyte expansion in monolayers *in vitro* toward ACI must be standardized with biochemical stimuli to avoid the process of dedifferentiation and achieve sufficient expansion with minimal subculture. The goal of this study was to evaluate the potential of PRP and HAS, in combination with variable oxygen tension, to minimize chondrocyte dedifferentiation while maintaining adequate proliferation rates during *in vitro* monolayer expansion.

## Materials and Methods

### Human Subjects

Human osteoarthritic cartilage was obtained from a total of six donors (72.83 ± 8.98 years) undergoing total knee replacement surgery. Human blood was collected from 16 healthy male and female volunteers after written informed consent. The local ethical commission approved the study protocol (approval no. GS4-EK-4/249-2013).

### Preparation of Platelet Derivatives

Whole blood was collected from 10 individual blood donors of 37.2 ± 9.6 years (mean ± SD) into 9-mL silicon-coated blood collection tubes (VACUETTE^®^ z serum clot activator, Greiner bio-one) and centrifuged at 1,770 *g* for 10 min. The resulting fibrin clot was removed from the tube, and a bottom portion containing the red blood cells was cut and discarded. The fibrin clot was then squeezed with a non-absorbable impermeable sterile material in a sterile Petri dish to extrude the HAS, and the HAS samples were stored at −80°C until further use. PRP was prepared by transferring whole blood from the same donors into 9-mL EDTA-coated blood collection tubes (VACUETTE^®^ K3EDTA, Greiner bio-one) and centrifuged at 440 *g* for 10 min. The platelet enriched plasma (middle layer) along the platelet poor plasma (supernatant) was transferred into a 15-mL falcon tube and centrifuged at 1,770 *g* for 10 min. The resulting pellets that contained an average of 10^7^ platelets/mL were resuspended in the superficial plasma layer corresponding to the volume obtained from the HAS in these individual donors. The PRP was stored at −80°C until further use.

### Growth Factor Evaluation

The composition of growth factors in HAS and PRP respective to individual donor variation were quantitatively determined for multiple human protein biomarkers, performed by a bead-based multiplex analysis. Human biomarkers basic fibroblast growth factor (bFGF), leptin, PDGF-BB, and G-CSF (Bio-RAD, Vienna, Austria) were used for this assay, according to the manufacturer’s instruction. Analyses of samples were performed with a Bio-Plex™ 200 system (Bio-RAD, Hercules, CA, USA).

### OA Chondrocyte Isolation

Cartilage tissues obtained from the six donors (72.83 ± 8.98 years) were washed in phosphate-buffered saline and minced. OA chondrocytes (OAC) were isolated by enzymatic digestion as previously described (Jeyakumar et al., 2016) and expanded in growth medium (GIBCO^®^ DMEM/F12 GlutaMAX™-I, Invitrogen, Vienna, Austria) containing 2.5 µg/mL Amphotericin B and 0.1 mg/mL streptomycin (Sigma, Steinheim, Germany) with either 10% FCS (PAA Laboratories GmbH, Linz, Austria). All assays were performed on passage 1 chondrocytes to minimize the extent of dedifferentiation over subcultures.

### Cell Proliferation Assay

OA chondrocytes were seeded onto the 96-well plates at a seeding density of 3,000 cells/cm^2^ and cultured in control medium (10% FCS) for 48 h for cells to adhere. Post 48-h medium was replaced to treatment conditions with 10% HAS or 10% PRP or 10% FCS in experimental groups. Heparin (2 U/mL) was added to cultures with PRP to prevent gelation. The influence of HAS or PRP on cell proliferation was measured by the XTT (sodium 3′-[1-(phenylamino carbonyl)-3,4-tetrazolium]-bis (4-methoxy-6-nitro) benzene sulfonic acid hydrate) assay (Cell Proliferation Kit II; Roche Diagnostics GmbH, Mannheim, Germany) according to the manufacturer’s instructions. Briefly, the XTT reagents were added to the cell culture medium and incubated for 4 h at 37°C. Post-incubation, absorbance was measured at 450 nm with a background reference wavelength of 690 nm using a plate reader.

### Reverse Transcriptase Quantitative Real-time PCR (RT-qPCR)

The transcriptional expression of chondrogenic-specific genes was investigated with a real-time quantitative reverse transcriptase polymer chain reaction (RT-qPCR), according to the manufacturer’s instructions (Roche). Total mRNA was extracted using the high pure RNA isolation kit (Roche Diagnostics GmbH, Mannheim, Germany). The mRNA was reverse transcripted with a first strand cDNA synthesis kit (Roche Diagnostics GmbH, Mannheim, Germany), and cDNA samples were examined for chondrogenic markers COL1A1, COL2A1, MMP3, and matrix metalloproteinase 13 (MMP13) with Random Primer p(dN)_6_ according to the manufacturer’s instruction with RT-qPCR analysis performed in a cycler. The amount of amplified endogenous GAPDH expression level was used as an external reference gene. A 2^−ΔΔCt^ method was used to evaluate the relative expression level of mRNA for each target gene (Livak and Schmittgen, 2001) (Table 1).

**Table 1:** Sequences of primers and conditions used in reverse transcriptase quantitative real-time PCR.

Target gene	Primer forward	Primer reverse
*GAPDH*	CTCTGCTCCTCCTGTTCGAC	ACGACCAAATCCGTTGACTC
*COL2A1*	GTGTCAGGGCCAGGATGT	TCCCAGTGTCACAGACACAGAT
*COL1A1*	GGGATTCCCTGGACCTAAAG	GGAACACCTCGCTCTCCAG
*MMP3*	CAAAACATATTTCTTTGTAGAGGACAA	TTCAGETATTCGCTTGGGAAA
*MMP13*	TTTCCTCCTGGGCCAAAT	GCAACAAGAAACAAGTTGTAGCC

### Statistical Analysis

Non-parametric Mann–Whitney *U*-test were used to make comparisons between paired data, and multiple comparisons were performed by non-parametric Friedman tests followed by Dunn’s *post hoc* test. All data are presented as the mean ± SD. Statistical significance was set at *p* < 0.05. All statistical analysis was performed using GraphPad Prism Software (Graphpad Prism Software Inc., San Diego, CA, USA).

## Results

### Determined Protein Levels in Individual HAS, PRP Donors, and Isolation Time Dissimilarities in Different PRP and HAS Donors

**Figure 1:**
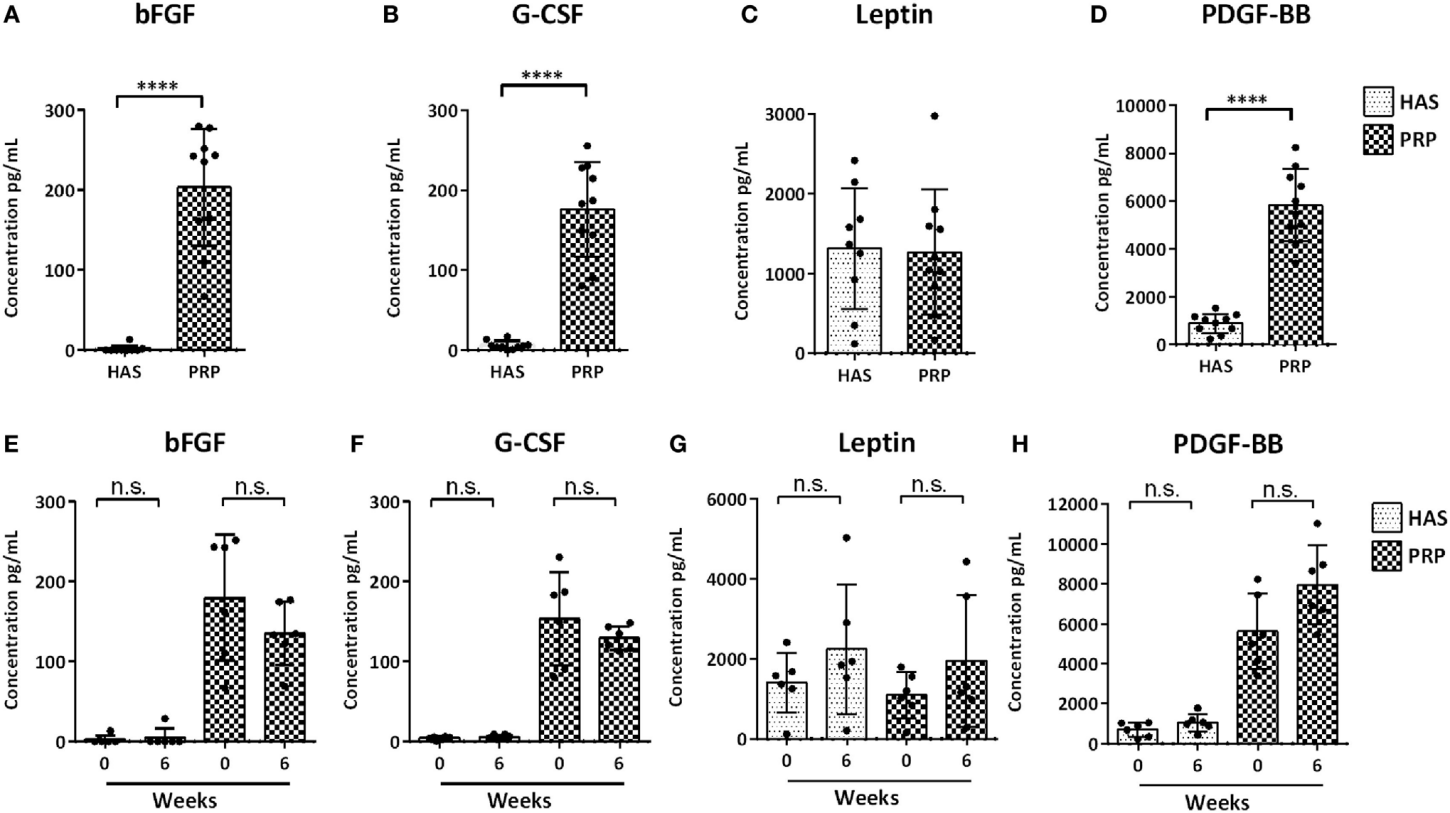
Analysis of determined protein content of hyperacute serum (HAS) and platelet-rich plasma (PRP) from 10 individual blood donors **(A–D)** and differences in protein levels isolated from 6 blood donors at 0 and 6 weeks for determining differences in protein levels to different isolation time points **(E–H)** determined by a multiplex bead-based array. Data represented as mean ± SD. Statistically significant is denoted by ****p < 0.0001.

Measured protein levels quantified from 10 individual blood donors revealed on average that bFGF was higher (Figure 1A) in PRP (20 ng/mL) than in HAS (0.18 ng/mL) (*p* < 0.0001). G-CSF was higher (Figure 1B) in PRP (17.6 ng/mL) than in HAS (0.61 ng/mL) (*p* < 0.0001). Leptin showed no significant differences (Figure 1C) between HAS (185.55 ng/mL) and PRP (126.73 ng/mL) (*p* = 0.782). PDGFF-BB was higher (Figure 1D) in PRP (582.66 ng/mL) than in HAS (89 ng/mL) (*p* < 0.0001). Similarly, HAS and PRP isolated at 0 and 6 weeks from six individual donors revealed that isolation time dissimilarities in the release kinetics of PDGF-BB, G-CSF, leptin, and bFGF (Figures 1E–H) were not significantly different among the individual donors (*p* = 0.1797, *p* = 0.4848, *p* = 0.5043, and *p* = 0.5887, respectively). The proteins quantified were not dependent on isolation time for either the HAS or PRP preparations.

### HAS, PRP from Individual Blood Donors on OA Chondrocyte Proliferation

**Figure 2:**
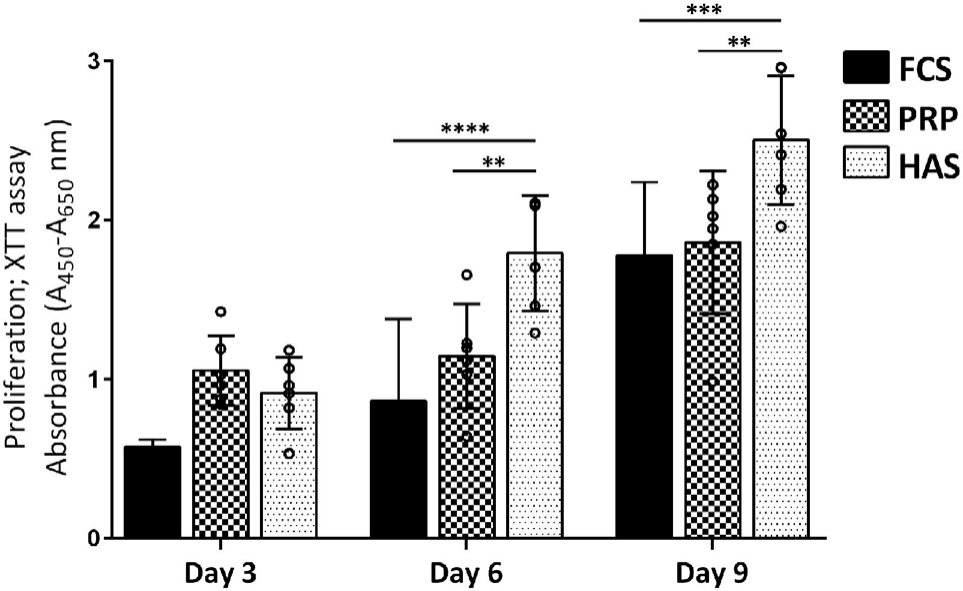
Effect of platelet-rich plasma (PRP) or hyperacute serum (HAS) from six individual blood donors on the proliferation of OA chondrocytes (OAC) from three donors. 10% HAS stimulated a higher response from all three donors of OAC on day 6, 9 compared with 10% FCS or PRP. Statistically significant is denoted by **p < 0.001 and ****p < 0.0001; *n* = 3 biological replicates.

Cell proliferation was determined based on the metabolic activity over 3, 6, and 9 days when cultured with 10% FCS, 10% PRP, or 10% HAS. No significant differences were observed over 3 days of culture among 10% FCS versus 10% PRP (*p* = 0.5045) or 10% FCS versus 10% HAS (*p* = 0.9425). The metabolic activity, however, significantly increased at day 6 in 10% HAS versus 10% FCS (*p* < 0.0001) and 10% PRP (*p* < 0.01). On day 9, 10% HAS enhanced the metabolic activity even higher versus 10% FCS (*p* < 0.001) and 10% PRP (*p* < 0.01) (Figure 2). Metabolic activity of the cells remained almost the same at all time points between 10% FCS and 10% PRP with no significant difference among them.

### Chondrogenic Gene Expression of OAC Cultured in Pooled HAS or PRP

**Figure 3:**
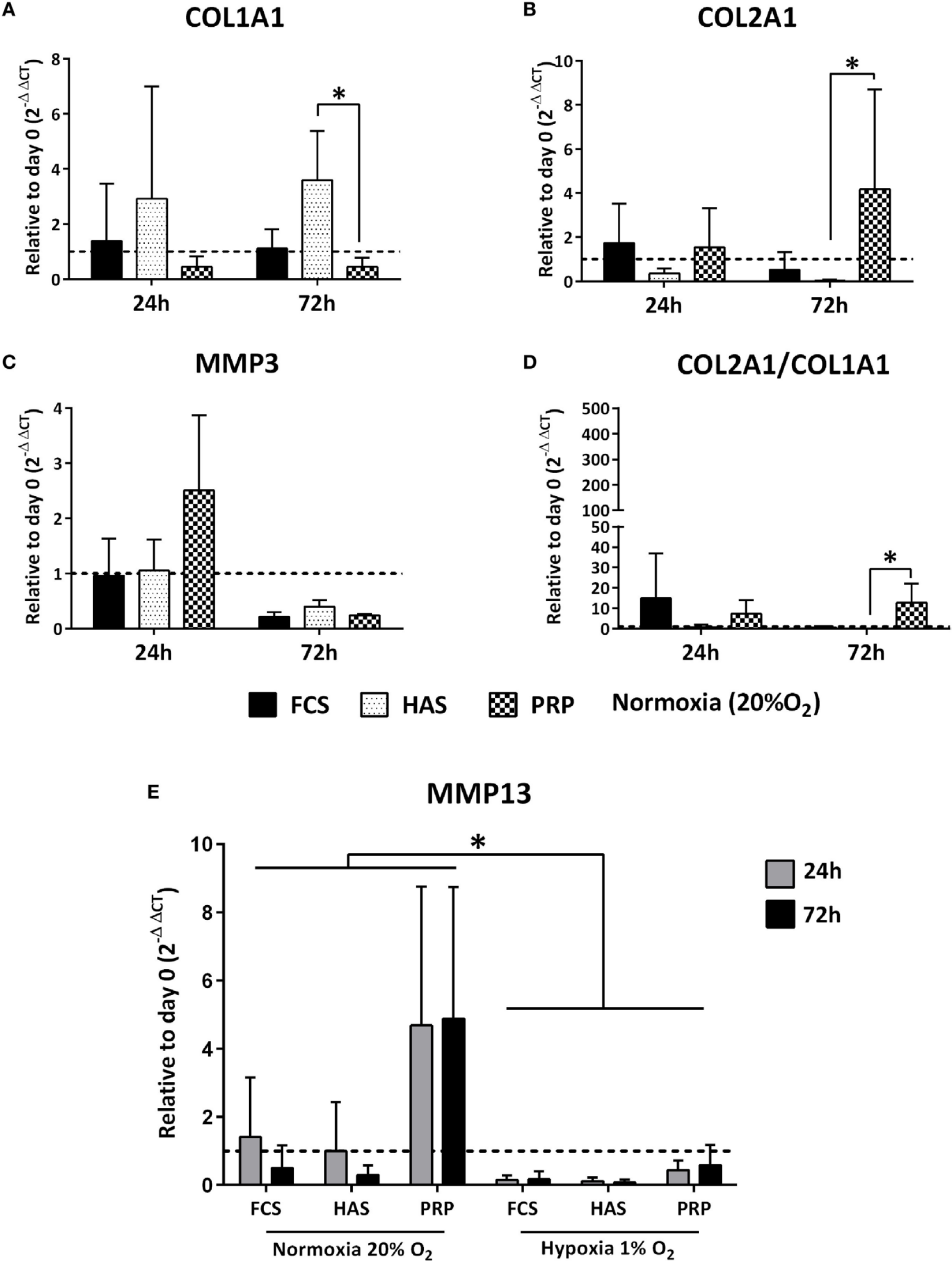
Differences in relative expression of chondrogenic markers COL1A1, COL2A1, MMP3 **(A–C)**, and the differentiation index **(D)** depicted to day 0 control under normoxic (20% O_2_) conditions. Matrix metalloproteinase 13 (MMP13) expression significantly downregulated under hypoxia (1% O_2_) than in normoxia (20% O_2_) **(E)** as determined by reverse transcriptase quantitative real-time PCR of OA chondrocytes cultured in FCS, hyperacute serum (HAS), or platelet-rich plasma (PRP). Significant difference at *p < 0.05; *n* = 3 biological replicates.

Quantitative RT-qPCR indicated that under normoxic conditions addition of 10% PRP led to an increased differentiation index (COL2A1/COL1A1) from a dedifferentiated phenotype within 24 and 72 h. 10% FCS led to a decrease in the index from 15-folds at 24 h to <1-fold at 72 h, and 10% HAS led to a decrease in the index both at 24 and 72 h compared to day 0 (Figure 3D). Under hypoxic conditions, the index for 10% PRP increased from 24 to 72 h. From 24 to 72 h, 10% FCS and 10% HAS exhibited similar decreases to the ones observed in normoxia (Figure S1 in Supplementary Material). This finding indicated that among the treatment conditions 10% PRP was capable of redifferentiating the dedifferentiated chondrocytes from day 0 to 72 h culture period (Figure 3A), while 10% FCS or 10% HAS led to further dedifferentiation (Figure 3B). No significant differences were observed in MMP3 under all conditions in both normoxia (Figure 3C) and hypoxia. MMP13 was downregulated from 24 to 72 h in 10% FCS and 10% HAS under both normoxic and hypoxic conditions. In 10% PRP, MMP13 was four times higher at both 24 and 72 h compared to day 0 under normoxia but downregulated under hypoxia (Figure 3E).

## Discussion

Our objective was to estimate the differences in growth factor levels among human PRP and HAS isolated from individual blood donors at different time points and examine their effect on chondrocyte proliferation in monolayers. We also examined the redifferentiation potential of dedifferentiated osteoarthritic chondrocytes. Our results suggest that growth factor levels do not differ among donors when isolated between any two of the pre-defined different isolation time points. HAS showed increases in chondrocyte proliferation versus PRP or FCS at the cost of dedifferentiation, but PRP has the potential to regenerate dedifferentiated chondrocytes on 2D substrates.

The PRP preparation used in our study contained six times more platelets than pre-prepared samples. PDGF-BB and LEPTIN were also found in higher amounts than bFGF or granulocyte colony-stimulating factor (GM-CSF) in PRP. These growth factor levels evaluated in our study have been studied for their underlying effect on chondrocytes in other studies. For example, GM-CSF has been reported to increase proliferation and anabolism in chondrocytes by inhibiting the IL-1β-induced catabolic effect (Quintero et al., 2008; Marmotti et al., 2013). GM-CSF has also been shown to induce proteoglycan, aggrecan, and collagen synthesis of articular chondrocytes (Quintero et al., 2001). PDGF-BB has also been able to enhance chondrocyte proliferation through the extracellular signal-regulated kinase 1/2 pathway (Xiao et al., 2014) and is active inhibitors of the IL-1β-mediated activation of NF-kB and apoptosis in chondrocytes (Montaseri et al., 2011). bFGF is believed to be a key regulator of maintaining the chondrocyte phenotype during expansion. bFGF has a catabolic effect on dedifferentiation by upregulating the matrix metalloproteinases MMP1, MMP13 and downregulating aggrecan, collagen II in human OAC (Nummenmaa et al., 2015). Treatment of chondrocytes with the adipokine leptin can induce anabolic effects such as proliferation, differentiation, and cytoskeletal remodeling (Liang et al., 2011) and could lead to a pro-inflammatory stimulus in interaction with IL-1 β-induced nitric oxide (NO) production. This further leads to loss of the differentiated phenotype, apoptosis, and degradation of the extracellular matrix (Kim et al., 2002; Otero et al., 2005). Based on the data mentioned earlier, we quantified these factors and hypothesized that PRP or HAS could stimulate proliferation and differentiation of dedifferentiated OAC.

Obtaining adequate cell numbers for ACI treatment of cartilage defects is achieved by *in vitro* expansion on 2D surfaces. During this expansion, chondrocytes lose the characteristic round morphology, and phenotypic change to fibroblasts occurs as part of dedifferentiation. We found that the effects of HAS on increased proliferation in comparison to PRP or FCS were consistently higher from six blood donors on three osteoarthritic chondrocyte donors. Accelerating sufficient cell numbers in short culture time could reduce the waiting time of transplantation for ACI procedures. However, such accelerated proliferation did not redifferentiate the chondrocytes when cultured with HAS rather more dedifferentiation accompanied by an increase in COL1A1 expression was observed. PRP did enhance proliferation on monolayer culture and at the same time induces redifferentiation by a decrease in COL1A1 and increased COL2A1 expression both under normoxic and hypoxic conditions. Spreafico et al. (2009) observed similar effects on increased anabolic gene expression, proliferation, and proteoglycan production when chondrocytes were cultured with PRP in comparison to human serum or FCS. Contrary to our study, some reports on PRP has been shown to have a detrimental effect on differentiation (Gaissmaier et al., 2005; Drengk et al., 2009) while others have reported a positive effect on differentiation (Akeda et al., 2006; Filardo et al., 2012). It may be that increased proliferation of cells leads to a decrease in type II collagen mRNA or *vice versa* in these studies as a cell cannot proliferate and differentiate at the same time. Recently, PRP’s effect on adipose-derived stem cells toward chondrogenesis has been positively shown to induce chondrogenic differentiation without addition of exogenous growth factors and differentiated cells secreting less angiogenic/inflammatory markers (Pakfar et al., 2017). A similar effect was observed when chondrocyte/MSC cocultures were cultured in the presence of PRP also by reducing the hypertrophic marker expression in cocultures (Ramezanifard et al., 2017). Several cytokines and signaling pathways involved in modulating chondrocyte dedifferentiation are reported as implications for cell-based cartilage therapies (Duan et al., 2015), and future interrogations should decipher and standardize these factors from PRP, HAS, and HS formulations.

Hypoxia was used to mimic the native environment in the current study, and we observed that low oxygen tension could inhibit the catabolic MMP13 expression during the redifferentiation process than in normoxia. Bouaziz et al. (2016) have shown that hypoxia-inducible factor α acts as a negative regulator of the Wnt/β-catenin signaling which in turn downregulates MMP13 expression and as a result reduced cartilage loss occurs. Although PRP formulations vary disparately, one of the important factors to be noted is the leukocyte-rich or leukocyte-poor PRP. The presence of high leukocyte content increased the inflammatory factors such as IL-1β and catabolic cytokines (Sundman et al., 2011) whereas low leukocyte content led to dedifferentiation (Gaissmaier et al., 2005). Taking these factors into account, overcoming dedifferentiation on 2D surfaces than accelerated *ex vivo* expansion should be of consideration for cell-based therapies such as ACI and MACI. ACI is, however, not recommended by the International Cartilage Repair Society as a therapeutic option for patients over 40–50 years or those who suffer from osteoarthritis. However, a recent follow-up clinical study investigating ACI for young patients (38.3 years) suffering from early stage of osteoarthritis has indicated this technique to be a potential treatment option for degenerative disorders (Minas et al., 2010).

## Conclusion

Our data indicate that HAS has its main effect on proliferation rather than redifferentiation while PRP enhances both proliferation and redifferentiaion of OAC during *ex vivo* expansion. Due to its autologous source and low cost, PRP has significant advantages over other therapies utilizing recombinant growth factors. The benefit of harvesting PRP from an individual patient offers a personalized formulation of a bioactive ECM milieu for enhancing chondrocyte culture conditions. We propose that OAC can be cultured with autologous PRP supplementation in growth medium for priming the cells for chondrogenic differentiation. This concept could be used in cell expansion for ACI procedures as a future improvement to existing therapies. Disadvantages include variability in plasma preparation protocols, differences in patients at various times of blood draws. HAS, however, enhances proliferation of OAC at the expense of increased dedifferentiation.

## Ethics Statement

This study was carried out in accordance with the recommendations of Land Niederösterreich with written informed consent from all subjects. All subjects gave written informed consent in accordance with the Declaration of Helsinki. The protocol was approved by the “Land Niederösterreich by approval no. GS4-EK-4/249-2013.”

## Author Contributions

SN acquired funding for this research. VJ, EN-M, and ZL conceived idea, designed experiments, analyzed data, and interpreted results. VJ, EN-M, and CB performed experiments. VJ, ZL, and SN drafted the manuscript. All authors reviewed, revised, and approved the final version of the manuscript.

## Conflict of Interest Statement

The authors declare that the research was conducted in the absence of any commercial or financial relationships that could be construed as a potential conflict of interest. The reviewer CG and handling editor declared their shared affiliation.
