# Supplementary material for: Platelet-Rich Plasma Supports Proliferation and Redifferentiation of Chondrocytes during In Vitro Expansion
Source: Front Bioeng Biotechnol. 2017 Dec 6;5:75. doi: 10.3389/fbioe.2017.00075 (PMC5723650; doi:10.3389/fbioe.2017.00075)
Supplement: Supplementary file 1 [file Image_1.PDF]

{Supplemental Figure 1}

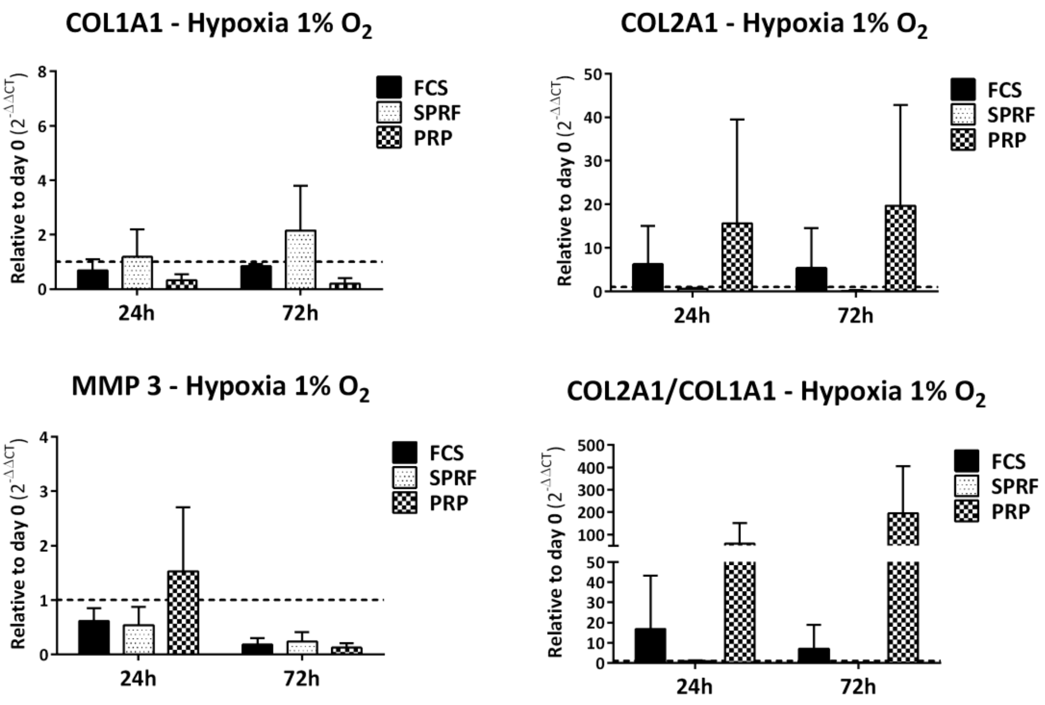

Supplement Fig. 1 Differences in relative expression of chondrogenic markers COL1A1, COL2A1, MMP3 (A-C) and the differentiation index (D) depicted to day 0 control under hypoxic (1%O_2) conditions as determined by RT-qPCR of OA chondrocytes cultured in FCS, HAS or PRP. Significant difference at *P <0.05; n=3 biological replicates.
